# Calix[4]arene-click-cyclodextrin and supramolecular structures with watersoluble NIPAAM-copolymers bearing adamantyl units: *“Rings on ring on chain”*

**DOI:** 10.3762/bjoc.6.83

**Published:** 2010-08-05

**Authors:** Bernd Garska, Monir Tabatabai, Helmut Ritter

**Affiliations:** 1 Institut für Organische Chemie und Makromolekulare Chemie, Heinrich-Heine-Universität Düsseldorf, Universitätsstraße 1, 40225 Düsseldorf, Germany

**Keywords:** β-cyclodextrin, calix[4]arene, click chemistry, poly(NIPAAM)

## Abstract

We describe the calixarene-cyclodextrin-coupling via click reaction starting from 5,11,17,23-tetra-*tert*-butyl-25,27-dipropargylether-26,28-hydroxy-calix[4]arene (calix[4]arene-dipropargylether) (**2**) onto 6I-azido-6I-deoxycyclomaltoheptaose (**3**) under microwave assisted conditions. The coupling was proven by MALDI-TOF mass spectrometry, ^1^H NMR and IR-spectroscopy. The pH dependent supramolecular complex formation with poly(NIPAAM) bearing attached adamantyl units was investigated by dynamic light scattering (DLS) and turbidity measurements.

## Introduction

Supramolecular interactions of macrocycles with different types of guest molecules are of increasing practical and theoretical interest [1–3]. In this context, we recently coupled cyclodextrin (CD) with cucubituril via a click reaction and investigated the special interactions with some suitable copolymers [4]. Because of their capability to form host–guest superstructures, CDs and calixarenes turned out to be very attractive not only as molecular receptors but also as building blocks for the construction of supramolecular architectures [5]. For that reason, we were encouraged to couple these two different types of macrocycles via click type reactions. Recent progress in the field of supramolecular chemistry is based on click chemistry, a versatile and powerful tool that permits the modular assembly of new molecular entities [6–7]. Both CDs as well as calixarenes have already been modified by click chemistry [8–14]. However, the coupling of calixarenes and β-CD via click reaction and their application in the field of supramolecular chemistry has not yet been reported. Herein, we describe the synthesis and complexation behavior of a dual type calix[4]arene-click-cyclodextrin (**4**) receptor by the cycloaddition of a dipropargylether of calix[4]arene (**2**) onto 6I-azido-β-CD (**3**) under microwave assisted conditions.

## Results and Discussion

### 

#### Synthesis and characterization of compound 4

**Scheme 1:**
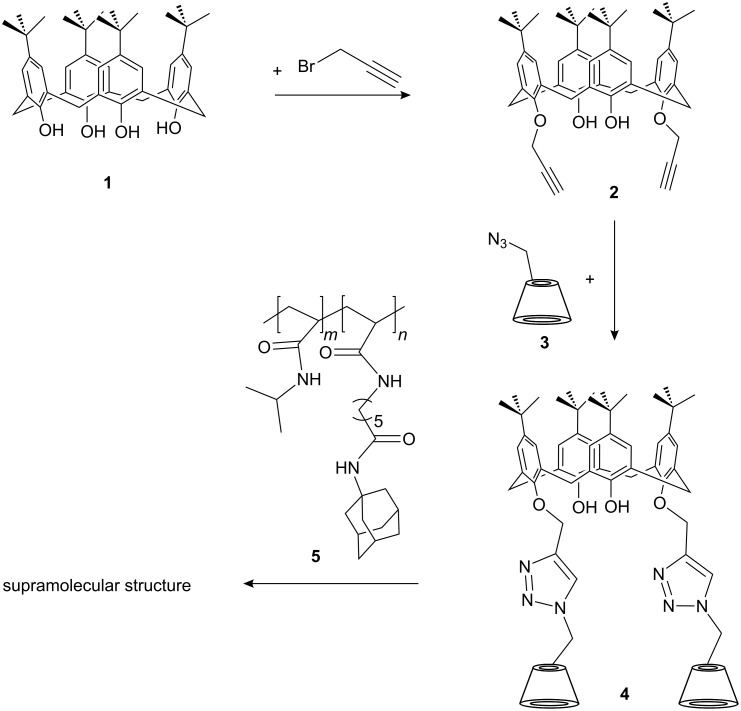
Synthesis of calixarene-click-cyclodextrin **4** via click chemistry and structure of copolymer **5**.

The calixarene-click-CD compound (**4**) was synthesized as shown in Scheme 1.

The successful microwave assisted cycloaddition of 5,11,17,23-tetra-*tert*-butyl-25,27-dipropargylether-26,28-hydroxy-calix[4]arene (calix[4]arene-1,3-dipropargylether) (**2**) onto 6I-azido-6I-deoxycyclomaltoheptaose (**3**) was proven by IR spectroscopy by the disappearance of the bands for the azide group at 2105 cm^−1^ and for the propargyl group at 2115 cm^−1^, whilst the formation of the triazole ring was confirmed by the appearance of a new band at 1654 cm^−1^. The structure of compound **4** was additionally confirmed by ^1^H NMR spectroscopy with the appearance of an olefinic proton signal at 8.07 ppm (–C=CH–N) and the disappearance of the characteristic propargyl proton signal at 2.50 ppm. Furthermore, the aromatic signals at about 7.0 ppm and the *tert*-butyl groups at 1.0 to 1.2 ppm indicates the presence of the calix[4]arene component. The CD was confirmed by the presence of the H2–H6 protons at about 3.3 to 3.6 ppm, the primary hydroxy group at 4.5 ppm and the secondary hydroxy groups at 5.7 ppm. The ^1^H NMR spectrum of the successful cycloaddition of **2** and **3** indicates that the di-substituted calix[4]arene **4** was the major product along with a little amount of the mono-substituted compound. In addition, the MALDI-TOF-MS clearly confirmed the existence of the covalently combined rings (**4**) with a molecular mass of [M + Na^+^] = 3066 *m/z* (1 calix[4]arene-click- 2 CD).

DLS measurements were performed to evaluate the hydrodynamic diameter of the prepared compounds. Surprisingly, the number averaged hydrodynamic diameter of **4**, which is about 150 nm in aqueous solution, which suggests the formation of aggregates. In comparison, the hydrodynamic diameter of β-CD in water is about 1.5 nm, and that of calix[4]arene-1,3-dipropargylether (**2**) in CHCl_3_ is only 0.64 nm. To reduce the agglomeration, compound **4** was deprotonated by dissolution in aqueous NaOH at pH 12. The negative charge was expected to cause intermolecular electrostatic repulsion. Accordingly, the hydrodynamic diameter of **4** decreased in NaOH solution from 150 nm to 9.0 nm, which can actually be attributed to the existence of trimers.

#### Host–guest complexion of 4 and 5

**Scheme 2:**
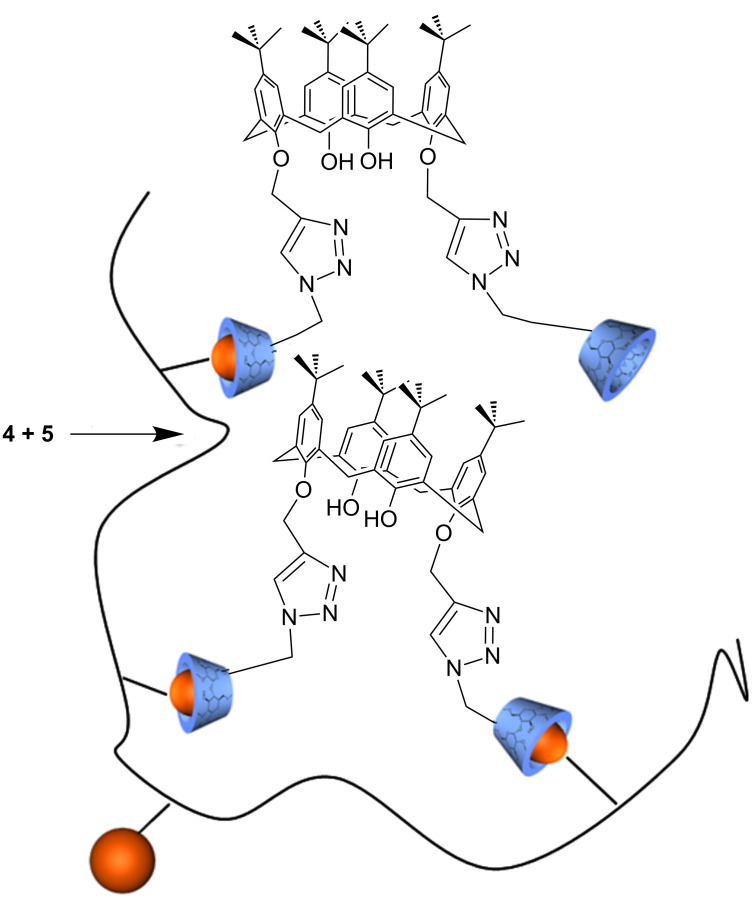
Superstructure of calixarene-click-cyclodextrin **4** and copolymer **5**.

An adamantane containing copolymer **5** was prepared via free radical polymerization of 6-acrylamido-*N*-adamantyl-hexane amide and NIPAAM. Copolymer **5** was mixed with **4** subsequently (Scheme 2) to form supermolecular structures.

The hydrodynamic diameter of copolymer **5** increased from 8.5 nm to 53 nm after addition of **4**. This clearly indicates the inclusion of a polymer attached adamantane moiety into the cavity of CD. The adamantane moiety is known to be one of the best guest molecules for β-CD [15]. A relatively high complex stability constant for a polymer attached adamantane groups with CD is about 5000 M^−1^ [16]. Therefore, as shown in Scheme 2, compound **4** obviously is expected to act as an intermolecular linker between the copolymer chains. To prove the assumed agglomeration of the CD-moieties in water [17], adamantyl carboxylate was added to the solution as a competitive guest molecule. As expected, a decrease of the hydrodynamic diameter from 53 nm to 13 nm was observed. Adamantyl carboxylate is known to be a more effective guest [15] than the polymer attached adamantane moiety itself. Thus, the inclusion of the low molecular weight adamantyl carboxylate into the cavity of the CD component of **4** leads to a replacement of copolymer **5**.

#### pH-Depending and LCST measurements of the host–guest complex

To prevent the agglomeration effects of the CD in compound **4** as discussed above, copolymer **5** and compound **4** were dissolved in an aqueous NaOH solution at pH 12 to deprotonate the free phenolic groups of the calix[4]arene-derivative **4**. After that, the hydrodynamic diameter of copolymer **5** increased due to complexation with negatively charged **4** from 8.5 nm to 17.5 nm. This increase of the hydrodynamic diameter again indicates the inclusion of the polymer attached adamantane moiety into the cavity of the CD-component. Comparing the diameters of complex (**4** + **5**) at pH 7 (53 nm) and at pH 12 (17.5 nm), it can be supposed, that due to deprotonation intermolecular electrostatic repulsion takes place which decreases the agglomeration in the system.

The turbidity point of copolymer **5** in water, 29 °C, is significant lower than that of the unmodified poly(NIPAAM) at 34 °C, which can be ascribed to the existence of the hydrophobic adamantyl units in the copolymer. The host–guest effect of the coupled rings **4** on the cloud point of copolymer **5** was evaluated by turbidity measurements. Only a slight positive shift of the cloud point temperature from 29 °C to 30 °C was found (Table 1). The temperature shift relative to the cloud point of poly(NIPAAM) itself, can be explained by the inclusion of the hydrophobic adamantyl units of **5** by the CD moiety of **4**, which, in principal, should increase the cloud point temperature. In contrast to this, the unavoidable presence of the hydrophobic calixarene units of **4** leads to a reduction of the cloud point temperature. Repeating the turbidity experiment at pH of 12, the cloud point temperature of the copolymer **5** decreased from the original 29 °C to 23 °C due to salt and pH effects [18]. However, after adding the calixarene-click-cyclodextrin (**4**) to copolymer **5** under similar conditions at pH 12, the cloud point increased to 30.5 °C. This increase of the cloud point can be explained by the deprotonation of the calixarene moiety of **4** to the corresponding phenolate structure, which causes an increase in hydrophilicity.

**Table 1:** Experimental cloud point temperature (LCST) and hydrodynamic diameter^a^ depending on the balance of hydrophobic/hydrophilic interactions of calixarene-click-cyclodextrin **4** with copolymer **5** depending on pH.

Compound number (pH)	Turbidity point (°C)	Number averaged hydrodynamic diameter (nm)

**4** (7)		150
**4** (12)		9.0
**5** (7)	29.0	8.5
**5** (12)	23.0	8.5
**5** + **4** (7)	30.0	53.0
**5** + **4** (12)	30.5	17.5

^a^DLS measurements were performed at 10 °C, below the turbidity point of the copolymer.

## Conclusion

A calixarene-click-cyclodextrin combi-receptor (**4**) was synthesized via copper-catalyzed Huisgen 1,3-dipolar cycloaddition. Investigations of the interactions with an adamantyl moiety containing copolymer have been carried out, showing the existence of a supramolecular structure. Due to deprotonation of the calixarene moieties at higher pH values, a significant change in solubility and hydrodynamic diameter was observed and correlated to the formation of superstructures.

## Experimental

**Materials.** β-Cyclodextrin (β-CD) was purchased from Wacker Chemie GmbH (Burghausen, Germany) and used after drying overnight over P_4_O_10_ under an oil pump vacuum. *N*-Isopropylacrylamide (NIPAAM) 97%, sodium azide (99.5%) and azobisisobutyronitrile (98%) were purchased from Aldrich Chemicals (Germany) and used as received. Copper-(II)-sulfate pentahydrate (99%) was obtained from Carl Roth GmbH & CO., and sodium L(+)-ascorbate (99%) obtained from AppliChem (Germany). *N*,*N*-Dimethylformamide (DMF) and sodium hydroxide (NaOH) were purchased from VWR (USA). Dimethylsulfoxide-*d_6_* 99.9% atom% D was obtained from Deutero GmbH (Germany). Commercially available reagents and solvents were used without further purification. calix[4]arene **1** [19], calix[4]arene-1,3-dipropargylether **2** [8], 6I-azido-6I-deoxycyclomaltoheptaose **3** [20] and poly(6-acrylamido-*N*-adamantyl-hexane amide-co-NIPAAM) **5** [4] (*M*_w_: 94900 g/mol, PDI: 3.5) were prepared according to methods described in literature.

**Measurements.** IR spectra were recorded with a Nicolet 6700 FTIR (Fourier transform infrared) spectrometer equipped with an ATR unit. The measurements were performed in the rage of 4000–300 cm^−1^ at room temperature. ^1^H NMR spectra were recorded on a Bruker Avance DRX 200 at 20 °C. Chemical shifts were referenced to the solvent value δ 2.51 for DMSO-*d*_6_. Matrix assisted laser desorption ionization time of flight mass spectrometry (MALDI-TOF-MS) was performed on a Bruker Ultraflex TOF mass spectrometer. Ions formed with a pulsed nitrogen laser (25 Hz, 337 nm) were accelerated to 25 kV, the molecular masses being recorded in linear mode. 2-(4-Hydroxyphenylazo)benzoic eacid (HABA) in DMF (25 mg/mL) was used as matrix. The samples (1 mg/mL in DMF) were mixed with the matrix solution at volumetric ratios of 1:10. Gel permeation chromatography (GPC) analyses were performed on a GPC system from PPS with PPS-WIN-GPC software 4.01, 6.1 with *N*,*N*-dimethylformamide as eluent. The flow rate was 1 ml min^−1^ and the column temperature was maintained at 60 °C. A 0.1% (w/w) polymer solution (100 µL) was applied to a hydroxyethyl methacrylate (HEMA) column combination that consisted of a precolumn of 40 Å and main columns of 40, 100 and 3000 Å porosities. The weight-average molecular weight (*M*_w_) and the polydispersity (PD) were calculated by a calibration curve generated by polystyrene standards with a molecular weight range from 370 to 1000000 Da. DLS experiments were carried out on a Malvern HPPS-ET apparatus at a temperature value of 10 °C. The particle size distribution was derived from a deconvolution of the measurement number averaged autocorrelation function of the sample by the general purpose mode algorithm included in the DTS software. Each experiment was preformed five times to obtain statistical information. Cloud points were determined by transmission changes (at 500 nm) of the solution heated at 1 K/min in a magnetically stirred cell; cloud points were defined as the temperature at which the transmission decreases by 50%. Microwave assisted synthesis was performed using a CEM Discover synthesis unit (monomode system). The temperature was measured by infrared detection with control and maintained at constant value by power modulation. Reactions were performed in closed vessels under controlled pressure.

**Synthesis of calix[4]arene-click-cyclodextrin 4.** The microwave assisted click reaction of calix[4]arene-1,3-dipropargylether (**2**) (100 mg, 0.14 mmol) with 6I-azido-6I-deoxycyclomaltoheptaose (**3**) (324.8 mg, 0.28 mmol) was carried out in DMF in the presence of Cu(I) generated by the reduction of copper sulfate (0.014 mmol) with sodium ascorbate (0.07 mmol). The tube was sealed, placed in the CEM monomode microwave and irradiated at 150 °C and 100 W for 30 min. The solvent was removed under reduced pressure. The crude product **4** was washed with water and dried in vacuum to afford a brown solid (yield: 70%).

MALDI-TOF: *m/z* 3066 [M + Na^+^]. ^1^H NMR (DMSO-*d*_6_, δ(ppm)): 1.05 (s, 9H, -C-(CH_3_)_3_), 1.13 (s, 9H, -C-(CH_3_)_3_), 1.17 (s, 9H, -C-(CH_3_)_3_), 1.19 (s, 9H, -C-(CH_3_)_3_), 3.37 (br, 28H, H-2,4), 3.66 (br, 56H, H-3,5,6), 4.48 (br, 7H, O-CH-O), 4.75 (br, 7H, O-CH-O), 4.85 (br, 12H, OH-6), 5.78 (br, 2H, Ar-OH), 6.97 (s, 2H, Ar-H), 7.01 (s, 2H, Ar-H), 7.06 (s, 2H, Ar-H), 7.10 (s, 2H, Ar- H), 7.78 (s, 1H, N-CH=C), 8.07 (s, 1H, N-CH=C). IR: 3328 (-OH), 2929 (aryl, alkyl), 1654 (triazole), 1482 (-C=N-), 1386 (-C(CH_3_)); further intensive signals, 1151, 1078, 1022 cm^−1^.

**Host–guest complexion of polymer 5 and calix[4]arene-click-cyclodextrin 4.** For further investigations, polymer **5** (50 mg, 5.3 × 10^−3^ mmol) was dissolved in 5 ml water or aqueous NaOH at pH 12. Product **4** (10 mg, 3.2 × 10^−3^ mmol) was added and the solution was mechanical stirred for 24 h. This solution was centrifuged to remove undissolved particles.
